# Involvement of ROS-alpha v beta 3 integrin-FAK/Pyk2 in the inhibitory effect of melatonin on U251 glioma cell migration and invasion under hypoxia

**DOI:** 10.1186/s12967-015-0454-8

**Published:** 2015-03-20

**Authors:** Cheng-Shi Xu, Ze-Fen Wang, Xiao-Dong Huang, Li-Ming Dai, Chang-Jun Cao, Zhi-Qiang Li

**Affiliations:** Department of Neurosurgery, Zhongnan Hospital of Wuhan University, Wuhan, 430071 PR China; Department of Physiology, School of basic medical science, Wuhan University, Wuhan, 430071 PR China; Department of Neurosurgery, Taihe Hospital of Shiyan, Shiyan, 442000 PR China; Laboratory of Neuro-oncology, Zhongnan Hospital of Wuhan University, Wuhan, 430071 PR China

**Keywords:** Melatonin, Glioma, Invasion, Migration, Hypoxia, Focal adhesion kinase

## Abstract

**Background:**

Melatonin, a well-known antioxidant, has been shown to possess anti-invasive properties for glioma. However, little is known about the effect of melatonin on glioma cell migration and invasion under hypoxia, which is a crucial microenvironment for tumor progress. In addition, focal adhesion kinase (FAK) and proline-rich tyrosine kinase 2 (Pyk2) are closely associated with cell migration and invasion. Therefore, we investigated the possible role of these kinases and its related signaling in the regulation of human U251 glioma cells behavior by melatonin under hypoxia.

**Methods:**

The abilities of migration and invasion of U251 glioma cells were determined by wound healing and transwell assay in vitro. The intracellular production of reactive oxygen species (ROS) was measured by using the fluorescent probe 6-carboxy-2′, 7′-dichorodihydrofluorescein diacetate (DCFH-DA). Immunofluorescence experiments and western blotting analysis were used to detect the expression level of protein. Small interfering RNAs (siRNA) was used to silence specific gene expression.

**Results:**

The pharmacologic concentration (1 mM) of melatonin significantly inhibited the migration and invasion of human U251 glioma cells under hypoxia. The inhibitory effect of melatonin was accompanied with the reduced phosphorylation of FAK and Pyk2, and decreased expression of alpha v beta 3 (αvβ3) integrin. Additionally, inhibition of αvβ3 integrin by siRNA reduced the phosphorylation of FAK/Pyk2 and demonstrated the similar anti-tumor effects as melatonin, suggesting the involvement of αvβ3 integrin- FAK/Pyk2 pathway in the anti-migratory and anti-invasive effect of melatonin. It was also found that melatonin treatment decreased the ROS levels in U251 glioma cells cultured under hypoxia. ROS inhibitor apocynin not only inhibited αvβ3 integrin expression and the phosphorylation levels of FAK and Pyk2, but also suppressed the migratory and invasive capacity of U251 glioma cells under hypoxia.

**Conclusions:**

These results suggest that melatonin exerts anti-migratory and anti-invasive effects on glioma cells in response to hypoxia via ROS-αvβ3 integrin-FAK/Pyk2 signaling pathways. This provides evidence that melatonin may be a potential therapeutic molecule targeting the hypoxic microenvironment of glioma.

**Electronic supplementary material:**

The online version of this article (doi:10.1186/s12967-015-0454-8) contains supplementary material, which is available to authorized users.

## Background

Melatonin, N-acetyl-5-methoxytryptamine, secreted predominately by the pineal gland, has antitumor properties on a variety of cancer types including glioma [1-3]. Millimolar concentrations of this indolamine were found to reduce U251 glioma cell growth by 70% after 72 hours of treatment, and intraperitoneal administration of melatonin (15 mg/kg body weight) to a rat subcutaneous U251 glioma model also reduced tumor growth by 50% [4]. In patients with glioblastoma, the most common primary malignant brain tumor, a strategy using radiotherapy plus melatonin resulted in an increase in survival compared with radiotherapy alone [5]. Moreover, combinations of melatonin and chemotherapeutic drugs also demonstrated a synergistic toxic effect on brain tumor stem cells [6]. Accumulative evidence further indicates the antitumor function of melatonin and its well-known antioxidant properties have been shown to be involved in the inhibitory effect on tumors. Melatonin and its metabolites are documented free radical scavengers and antioxidants [7]. Millimolar concentrations of melatonin displayed the ability to inhibit glioma cell migration and invasion through the inhibition of the oxidative stress pathway [1,2]. However, the majority of those studies were performed under normoxia, and little was done to observe the effect of melatonin on glioma under hypoxia, which results in the change of intracellular reactive oxygen species (ROS) status and is also a crucial microenvironment for tumor progress.

It is well documented that rapid tumor growth and insufficient blood supply leads to intratumoral hypoxia. Hypoxia is a key factor for the modulation of the biological behavior of glioma cells during tumor development [8]. The presence of hypoxic areas in glioblastoma is also considered to be an important determinant in tumor response to therapy, particularly to radiotherapy [9]. When tumor cells are exposed to hypoxia, ROS are increased and many functional genes that play important roles in glioma angiogenesis and tumor cell migration or invasion are upregulated or downregulated [10]. Recently, emerging evidence suggests the involvement of ROS and the aberrant activation of redox-sensitive signaling pathways in tumor invasion and migration [11,12]. Given the increased level of intracellular ROS under hypoxia and the marked antioxidant effect of melatonin, melatonin treatment might be a promising strategy for the modulation of hypoxia-related glioma cell biological behavior via the regulation of the intracellular ROS level. However, the ROS-related signaling involved in the action of melatonin on glioma under hypoxic conditions has not been well investigated so far.

Some ROS-regulated proteins were identified to play key roles in epithelial-mesenchymal transition and tumor metastasis. Integrin family members are of those ROS-regulated proteins [13,14]. Integrins are heterodimeric cell surface glycoproteins consisting of α and β subunits and control the attachment of cells to the extracellular matrix (ECM). Cell migration and invasion depend on the binding of integrins to the ECM, which leads to the recruitment of focal adhesion kinase (FAK) and/or proline-rich tyrosine kinase (Pyk2) to the newly formed focal adhesion sites [15]. Then, the activation of FAK and Pyk2 is followed by the phosphorylation of a variety of downstream effectors, resulting in cell migration and invasion [16,17]. Besides cell adhesion and migration, different heterodimeric integrin molecules mediate various complex processes including angiogenesis in a cell type- and context-dependent manner [18]. The expression of β3 integrin is mainly associated with tumor metastasis, and the αvβ3 heterodimer has been implicated in the malignant behavior of various tumor types, including glioma, melanoma, breast, and ovarian cancer [19-21]. Previous limited researches also demonstrated the possible effect of melatonin on cellular integrins expression and FAK activation. In MCF-7 human breast cancer cells, melatonin could shift it to a lower invasive status by increasing the β1 integrin subunit expression [22]. In umbilical cord blood-mesenchymal stem cells, melatonin was demonstrated to trigger FAK/paxillin phosphorylation to stimulate reorganization of the actin cytoskeleton [23]. Despite the important role of αvβ3 integrin and FAK/Pyk2 in glioma cell motility and the anti-invasive effect of melatonin, it remains unknown whether melatonin influences αvβ3 integrin expression and FAK/Pyk2 activation in glioma cells.

Taken together, it is intriguing to profoundly explore whether melatonin inhibits glioma cell migration and invasion under hypoxia via regulating ROS level, consequently modulating αvβ3 integrin expression and the activation of FAK and Pyk2. The present work displayed the inhibitory effect of melatonin at pharmacologic concentrations on glioma cell migration and invasion under hypoxia. A novel ROS-αvβ3 integrin-FAK/PyK2 pathway was shown,for the first time, to involve in the inhibitory effect of melatonin on U251 glioma cell migration and invasion. The present study, therefore, amplified the antitumor mechanisms of melatonin and provided a basis for its potential therapeutic application of malignant glioma.

## Methods

### Cell culture

Human glioma cells (U251) were obtained from Chinese Type Culture Collection (Chinese Academy of Sciences, Shanghai). The U251 glioma cell line was cultured in DMEM with 10% fetal bovine serum (FBS), 2 mM L-glutamine, 100 U/ml penicillin and 100 μg/ml streptomycin (Invitrogen Gibco, USA) at 37°C under 5% CO_2_ humidified air. All experiments were conducted using 80–85% confluent cells. Before each experiment, the plated cells were incubated with serum-free medium for 6 hours. Then, the medium was replaced with serum-free DMEM containing different concentrations of melatonin (Sigma-Aldrich, St Louis, MO, USA) under normoxic (20% O_2_) or hypoxic (1% O_2_) conditions. For hypoxia treatment, the cells were incubated in in hypoxic chambers (1% O_2_, 5% CO_2_, and 94% N_2_, Sanyo).

### Small interfering RNAs (siRNA) treatment of cells

For silencing specific gene expression, U251 glioma cells were treated with FAK siRNA, Pyk2 siRNA and integrin β3 siRNA (Santa Cruz Biotechnology, USA). Briefly, 2 × 10^5^ U251 glioma cells were seeded into six well plate with 2 ml antibiotic-free normal growth medium containing FBS. Transfection of FAK siRNA, Pyk2 siRNA, integrin β3 siRNA or control siRNA was performed according to the manufacture’s protocol. U251 glioma cells transfected with siRNA for 24 h were then exposed to hypoxia.

### Flow cytometry analysis of intracellular reactive oxygen species

The intracellular production of ROS was measured by using the fluorescent probe 6-carboxy-2′, 7′-dichorodihydrofluorescein diacetate (DCFH-DA). After 24 hours of treatment with melatonin at varied concentrations, cells were incubated with 10 μM DCFH-DA in serum-free medium for 10 minutes at 37°C. Afterwards, cells were harvested and resuspended in 500 μl of PBS, and DCF fluorescence was measured by a Beckman Coulter flow cytometer.

### Wound healing assay

The wound healing method described previously was used to assay cell migration ability [17,24]. In brief, 5 × 10^5^ U251 glioma cells were seeded into 6-well plates. An artificial homogenous wound was made with a sterile plastic 200 μL micropipette tip. After wounding, cell debris was removed by washing the cells with warm serum-free medium. After incubation with serum-free medium with or without melatonin for another 24 hours, cells that had migrated into the wounded area or with extended protrusion from the border of the wound were quantified after being photographed with an inverted microscope (40 × magnifications, Olympus, Japan). Data were from 5 independent experiments.

### Transwell migration and invasion assays

The in vitro migratory and invasive ability of glioma cells was assessed using the transwell chamber method [25]. In brief, U251 glioma cells were seeded into 24-well transwells (Corning Corp. USA) at a density of 5 × 10^5^ cells in 200 μL of medium in the upper chamber and were incubated in serum-free medium with or without melatonin, and the bottom chamber was filled with 600 μL of medium containing 10% FBS. After 24 hours of incubation at 37°C, non-migrating cells on the upper surface of the membrane were scrubbed gently with a cotton-tipped swab. The migratory cells on the lower surface of the membrane were fixed with 95% methanol and stained with 0.1% crystal violet (Sigma-Aldrich, MO, USA). Stained migratory cells were photographed under an inverted light microscope and quantified by manual counting in five randomly selected areas of view. Five independent experiments were performed. For invasion assays, U251 glioma cells were seeded into diluted matrigel-precoated 24-well transwells (Corning Corp. USA) at a density of 5 × 10^5^ cells in 200 μL of medium in the upper chamber. The procedure of cell treatment and staining was similar with transwell migration assay.

### Western blotting analysis

U251 glioma cells were rinsed in PBS and lysed with RIPA buffer (50 mmol/L Tris–HCl, pH 7.2, 150 mmol/L sodium chloride, 1% Nonidet P-40, 0.5% sodium deoxycholate, 0.1% sodium dodecyl sulfate) containing a protease and phosphate inhibitors after treatment for 12 hours. Western blotting was performed to detected protein expression and its phosphorylation statues by using specific antibodies against β-actin (1:2000), FAK (1:2000), phosphorylated FAK (Tyr397, 1:1000), Pyk2 (1:1000) or phosphorylated Pyk2 (Tyr402, 1:1000) [17]. All of these antibodies were purchased from Santa Cruz Biotechnology (USA). The protein bands were quantitatively analyzed by Kodak Digital Science ID software (Eastman Kodak Company, USA). The total protein level of FAK and Pyk2 was normalized to the expression of β-actin. The relative phosphorylation level of FAK and Pyk2 was normalized with corresponding total protein level.

### Immunofluorescence experiments

Expression of αvβ3 integrin protein in U251 glioma cells was examined via the immunofluorescence technique. U251 glioma cells on glass coverslips were incubated with medium containing different concentrations of melatonin under normoxia or hypoxia for 24 hours and were then fixed with 4% paraformaldehyde in PBS for 15 minutes at room temperature. Cells were then incubated with primary antibodies to αvβ3 integrin (1:100, Cell Signaling) overnight at 4°C. Binding specificity was controlled by an IgG isotype control (Jackson Immunoresearch). Subsequently, secondary antibodies Cy3-conjugated AffiniPure donkey anti-rabbit IgG (Jackson Immunoresearch) at 1/500 dilution were applied for 1 hour and viewed using a confocal microscope (Zeiss). αvβ3 integrin staining was presented as the average fluorescence intensity of three pictures per group. All sections were performed in Vectashield Mounting Media with 4′,6-diamidino-2-phenylindole (DAPI).

### Statistical analysis

All of the values are presented as the means ± S.E.M. Statistical analysis included *Student’s t* test analysis for 2 groups or one-way ANOVA for multiple groups comparisons. Differences were considered to be statistically significant at *p* < 0.05.

## Results

### Melatonin inhibits U251 glioma cell migration and invasion under hypoxia

**Figure 1:**
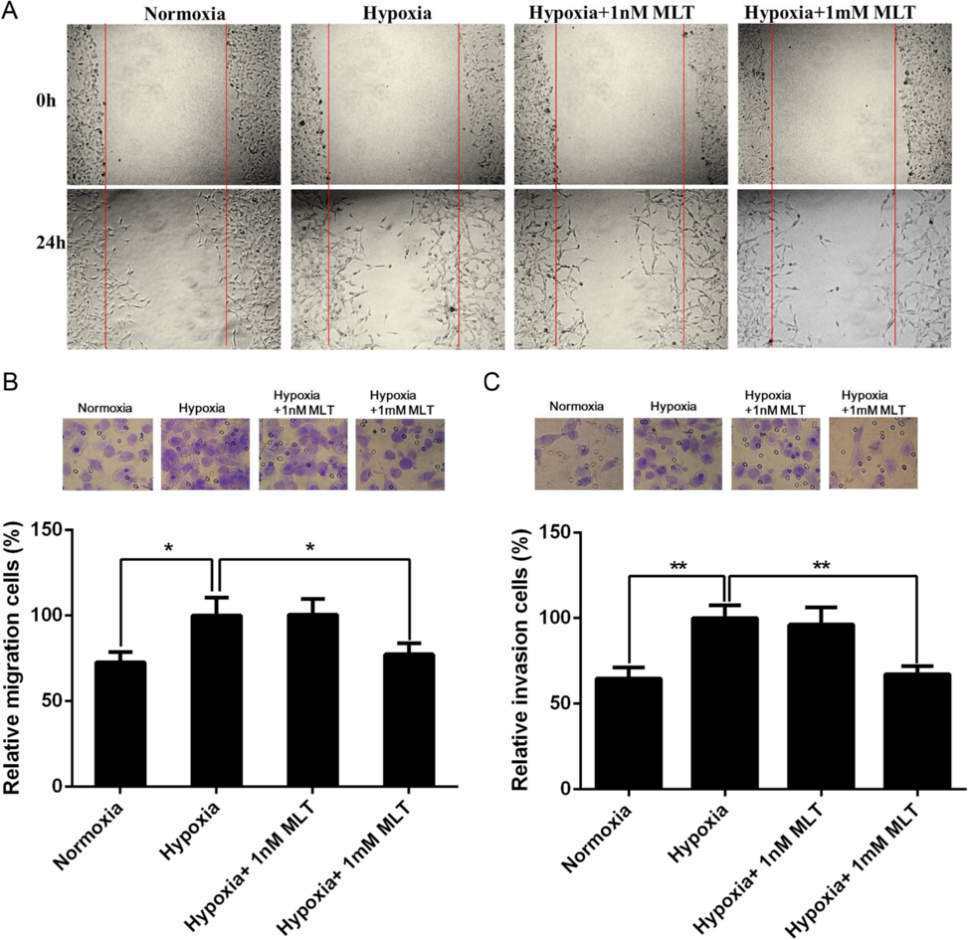
**Effect of melatonin on the migration and invasion of U251 glioma cells under hypoxia. (A)** The migratory ability of U251 glioma cells treated with different concentration of melatonin was evaluated by wound healing assay. The representative images at 0 hour and 24 hours post-wounding are shown at 10 × magnification. **(B)** U251 glioma cell migration was analyzed using transwell assay after melatonin treatment for 24 hours. The migratory cells were stained by 0.1% crystal violet (upper). The migratory cell number exposed to hypoxia alone was expressed as 100% (lower). **(C)** U251 glioma cell invasion was detected by matrigel-precoated transwell assay after melatonin treatment for 24 hours. The invasive cells were stained by 0.1% crystal violet (upper). The invasive cell number exposed to hypoxia alone was expressed as 100% (lower). The data presented the mean of three independent experiments. *p < 0.05, **p < 0.01.

Hypoxia, a common and important characteristic of the glioma microenvironment, has been shown to promote glioma cell migration and invasion. To evaluate the effects of melatonin on cell migration under hypoxia, wound healing and a transwell migration assay were performed. Compared with cells cultured under normoxia, many more U251 glioma cells migrated into the wound area under hypoxia in the wound healing assay, and simultaneous exposure to 1 mM melatonin significantly reduced hypoxia-induced U251 glioma cell migration (Figure 1A). However, melatonin at a physiological concentration (1nM) had no obvious effect on U251 glioma cell migration under hypoxia (Figure 1A). The inhibitory effect of melatonin on U251 glioma cell migration was further quantitatively confirmed by a transwell assay (Figure 1B). Similar results were also observed in the transwell invasion assay (Figure 1C). These results demonstrated that melatonin at pharmacological concentration could significantly inhibit U251 glioma cell migration and invasion under hypoxia.

### Melatonin modulates phosphorylation state of FAK and Pyk2 in U251 glioma cells

**Figure 2:**
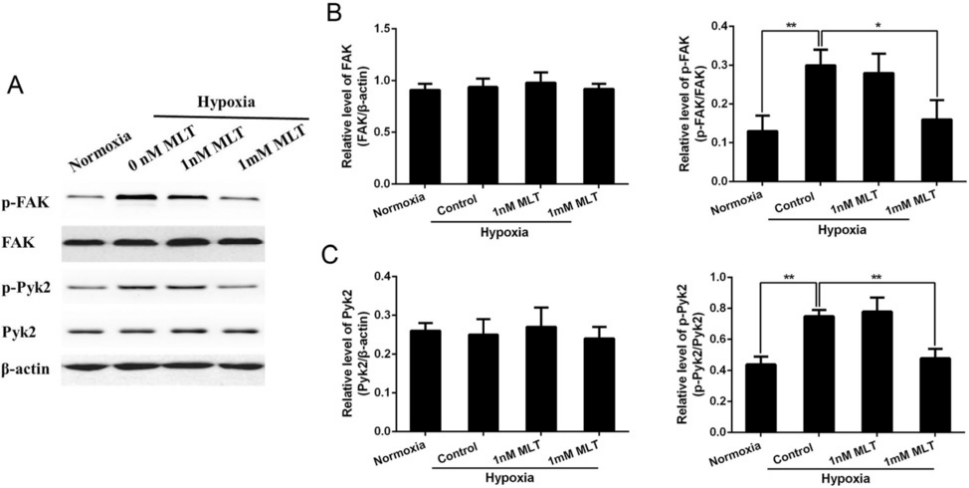
**Melatonin inhibited the phosphorylation of FAK and Pyk2 in cultured U251 glioma cells. (A)** The total and phosphorylation levels of FAK and Pyk2 in U251 glioma cells were determined by western blot analysis after different treatment for 12 hours. **(B, C)** The quantification of total and phosphorylated FAK and Pyk2 was analyzed respectively. *p <0.05, **p <0.01.

To explore the mechanism underlying the inhibitory effects of melatonin on cell migration and invasion, changes in FAK and Pyk2 activities were studied due to their important role in the formation of focal adhesions, which is a key process in cell migration and invasion. Tyr397 is an autophosphorylation site of FAK that triggers downstream events leading to cell migration. Activation of Pyk2 is indicated by an increase in its phosphorylation at Tyr402 [16]. Therefore, the phosphorylation state of FAK at Tyr397 and Pyk2 at Tyr402 was examined by immunoblotting. Melatonin treatment had no effect on total protein level of FAK and Pyk2 (Figure 2A). Then we evaluated the relative phosphorylation level of FAK and Pyk2 to the total protein level respectively. Compared with cells under normoxia, a significant increase in phosphorylation of FAK at Tyr397 and Pyk2 at Tyr402 was observed under hypoxia accompanied by comparable total protein levels (Figure 2B,C). The administration of 1 mM melatonin significantly reduced the phosphorylation level of both FAK and Pyk2 under hypoxia (Figure 2B,C). Similar results were also observed in U251 glioma cells after melatonin treatment under normoxia (see Additional file 1).

### Silencing of FAK and Pyk2 inhibit U251 glioma cells migration and invasion under hypoxia

**Figure 3:**
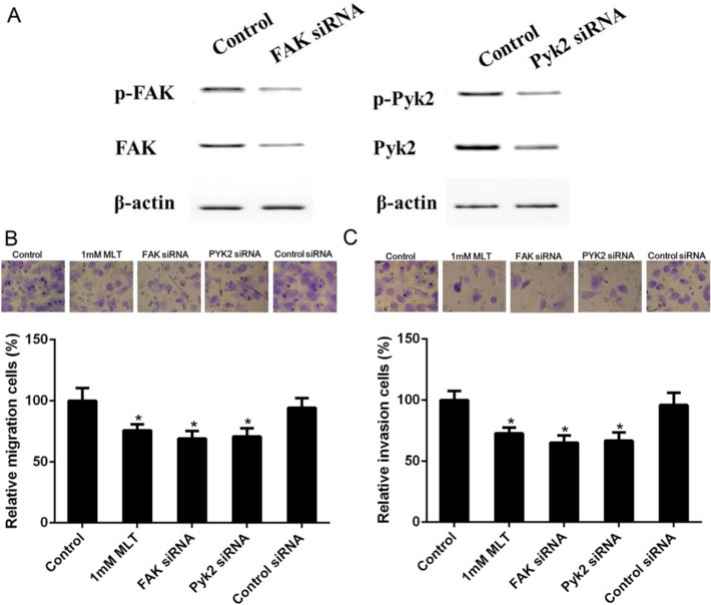
**Specific siRNA of FAK and Pyk2 inhibited hypoxia-induced U251 glioma cells migration and invasion. (A)** Specific siRNA significantly reduced the total and phosphorylation level of FAK and Pyk2. **(B)** Effect of FAK and Pyk2 siRNA on migration of U251 glioma cells using a transwell assay. The migratory cells were stained by 0.1% crystal violet (upper). The migratory cell number of control was expressed as 100% (lower) *p <0.05, **p <0.01. **(C)** Effect of FAK and Pyk2 siRNA on invasion of U251 glioma cells by a matrigel-precoated transwell assay. The invasive cells were stained by 0.1% crystal violet (upper). The invasive cell number of control was expressed as 100% (lower). *p <0.05, **p <0.01 *vs* control.

To investigate the involvement of FAK and Pyk2 in hypoxia-induced U251 glioma cell migration and invasion, specific siRNA of FAK and Pyk2 were applied to U251 glioma cells. As shown in Figure 3A, the efficiency of specific siRNAs was confirmed by significantly decreased total protein level of FAK and Pyk2. Phosphorylated FAK and Pyk2 were also reduced. It was shown that the specific siRNA of both FAK and Pyk2 significantly inhibited the migration (Figure 3B) and invasion (Figure 3C) of U251 glioma cells cultured under hypoxia, which was similar to the effect of the pharmacologic concentration of melatonin. These data suggested that melatonin may inhibit U251 glioma cell migration and invasion through the inhibition of FAK and Pyk2 activity.

### Attenuation of αvβ3 integrin expression mediates the effect of melatonin on FAK and Pyk2 phosphorylation

**Figure 4:**
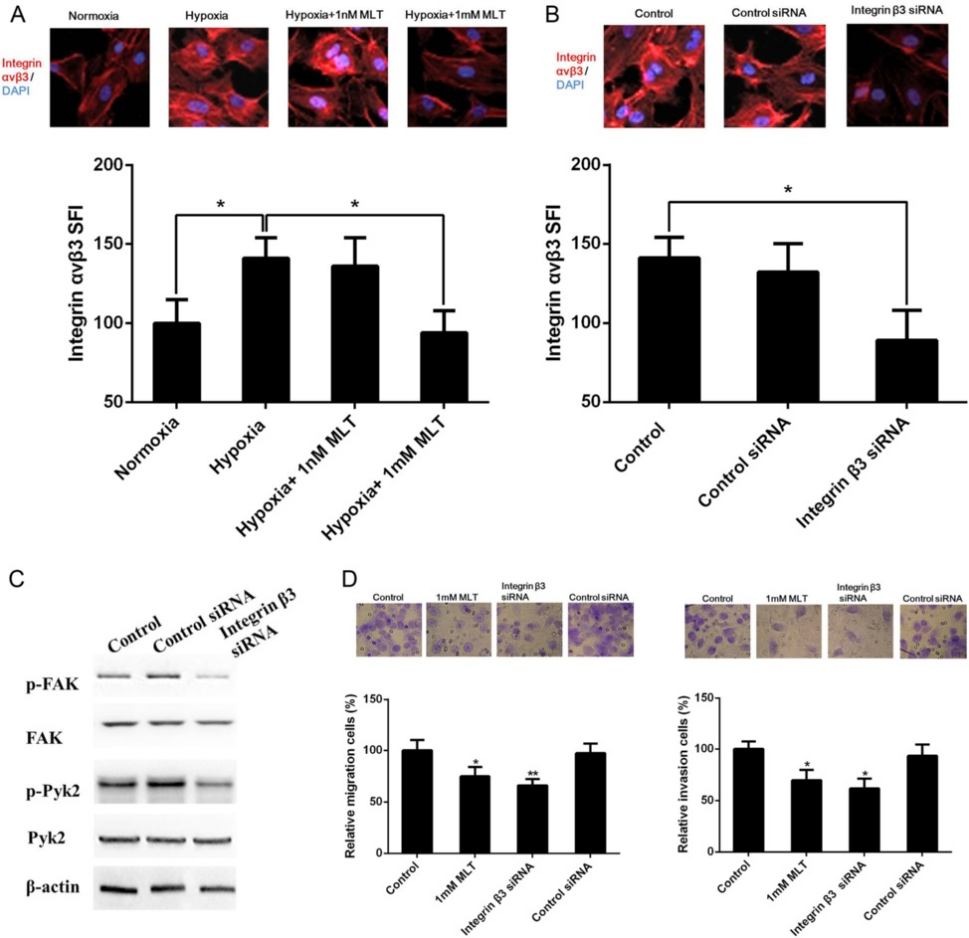
**Hypoxia required the** α**v**β**3 integrin pathway to drive the migration and invasion of U251 glioma cells.** The expression of αvβ3 integrin was determined by immunofluorescence after treatment with melatonin **(A)** or integrin β3 siRNA **(B)** under hypoxia. All data were expressed as specific fluorescence index (SFI) values. **(C)** Integrin β3 siRNA treatment reduced the phosphorylation of FAK and Pyk2 in U251 glioma cells under hypoxia. **(D)** Integrin β3 siRNA treatment inhibited the migration and invasion of U251 glioma cells under hypoxia. Cells were stained by 0.1% crystal violet (upper) and the cell number of control was expressed as 100% (lower). *p <0.05, **p <0.01 *vs* control.

It is widely known that focal adhesion kinases are mediators of the integrin pathway and that its phosphorylation can be activated by αvβ3 integrin under hypoxia in glioma cells; therefore, we determined whether the inhibitory effect of melatonin on FAK and Pyk2 phosphorylation might be mediated through αvβ3 integrin. We first examined αvβ3 expression and cellular localization in U251 glioma cells by immunofluorescence analysis. As shown in Figure 4A, hypoxia treatment caused a significant increase of αvβ3 integrin staining in U251 glioma cells. However, αvβ3 integrin staining was significantly decreased after the application of 1 mM melatonin under hypoxic conditions. Similar effect of melatonin on αvβ3 integrin was also observed under normoxia (see Additional file 2). These results strongly suggested that hypoxia might activate αvβ3 integrin by enforcing their membrane recruitment and that melatonin can attenuate the stimulation of αvβ3 integrin by hypoxia in U251 glioma cells.

Then, we used siRNA directed against the β3 subunit to explore the involvement of αvβ3 integrin inhibition on FAK and Pyk2 phosphorylation in U251 glioma cells under hypoxia. As shown in Figure 4B and C, αvβ3 integrin staining in U251 glioma cells was significantly reduced by specific siRNA and the levels of phosphorylated FAK at Tyr397 and phosphorylated Pyk2 at Tyr402 were also decreased after siRNA treatment. Consistent with this observation, the inhibition of αvβ3 integrin by siRNA also significantly inhibited U251 glioma cell migration and invasion, similar to the pharmacologic concentration of melatonin under hypoxia (Figure 4D). These results suggested that melatonin-inhibited migration and invasion involved the αvβ3 integrin and FAK/Pyk2 complex.

### Inhibition of ROS by melatonin involves its regulation to αvβ3 integrin

**Figure 5:**
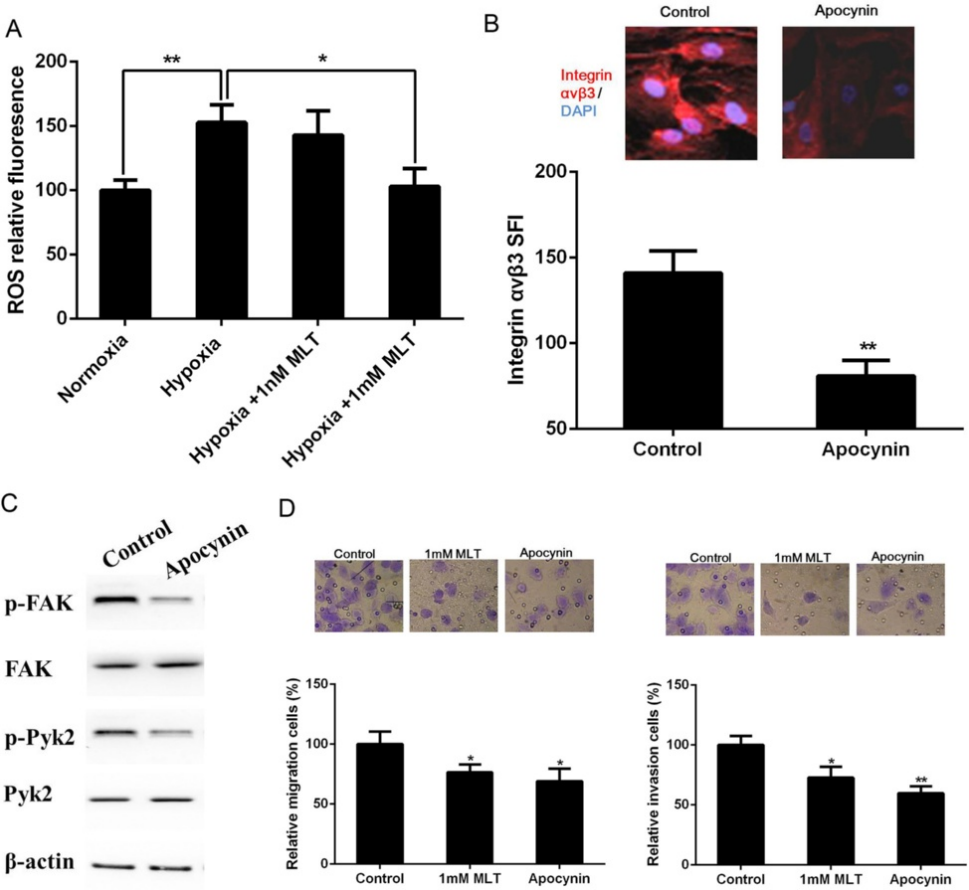
**Effect of ROS on migration and invasion of U251 glioma cells under hypoxia. (A)** Intracellular production of ROS were measured by flow cytometry analysis. The ROS inhibitor apocynin reduced the expression of αvβ3 integrin **(B)**, the phosphorylation of FAK and Pyk2 **(C)**, as well as the migration and invasion **(D)** of U251 glioma cells under hypoxia. *p <0.05, **p <0.01 *vs* control.

Melatonin is an effective free radical scavenger and possesses antioxidant effects. Furthermore, intracellular ROS is elevated under hypoxia and the oxidative stress pathway has been found to be closely associated with cell invasion. Thus, we evaluated the possible involvement of the antioxidant effects of melatonin on its anti-migration and anti-invasive properties by detecting the levels of intracellular ROS. Compared with normoxia, intracellular ROS was elevated under hypoxia. One millimolar melatonin significantly decreased the intracellular status of ROS under hypoxia (Figure 5A) and normoxia (see Additional file 3). However, DCF fluorescence was not changed by the physiological concentration (1 nM) of melatonin.

We next determined whether the regulation of αvβ3 integrin expression and FAK/Pyk2 phosphorylation by melatonin is mediated by its antioxidant effect. Remarkably, treatment with the ROS inhibitor apocynin (a NADPH oxidase inhibitor) significantly reduced the migratory and invasive capacity of U251 glioma cells under hypoxia in parallel to the pharmacologic concentration of melatonin. Exposure of U251 glioma cells to apocynin also inhibited αvβ3 integrin expression and the levels of phosphorylated FAK at Tyr397 and phosphorylated Pyk2 at Tyr402 (Figure 5B-D).

## Discussion

The hypoxic microenvironment is frequently found in solid tumors and contributes to the development of an aggressive and poor prognostic phenotype with high metastatic rates and chemo- or radio-resistance [26]. Glioma is the most common primary malignant tumor in the central nervous system (CNS) and the most aggressive brain tumor with very poor prognosis and is characterized by invasive growth, high vascularization with immature or abnormal vessels, and a recurrent tendency [27]. Due to the inefficient microcirculation in malignant glioma and the poor maintenance of the blood–brain barrier, malignant gliomas are more prone to chronic hypoxia [28]. Hypoxia is a well-characterized component of the malignant glioma microenvironment and has been demonstrated to promote cell invasion and migration [9,24,29,30]. It has been reported that hypoxia results in an increased generation of ROS, which are important mediators of the hypoxia-induced cellular response [31]. Several studies have suggested a close relationship between ROS and tumor cell invasion and migration [32,33]. The present in vitro data demonstrated that hypoxia stimulated migration and invasion in U251 glioma cells, and the levels of intracellular ROS were elevated under hypoxia. When the levels of intracellular ROS were reduced by its inhibitor apocynin, glioma cell migration and invasion were also inhibited. These consistent results suggest that targeting the elevated intracellular ROS is a possible strategy for the suppression of glioma cell migration and invasion.

Melatonin, a small lipophile, exhibits a variety of biological functions through either binding with its membrane receptors or the direct antioxidant effects [34,35]. Physiologically, melatonin is secreted at low nanomolar concentrations and a variety of its physiological functions are mediated mainly through a family of guanidine triphosphate-binding proteins or G protein-coupled receptors. The high-affinity melatonin receptors, type 1A and type 1B, share a close pharmacological profile and are activated by melatonin at nanomolar concentration [4,36,37]. Melatonin is also a well-documented antioxidant compound at pharmacological concentrations, which is almost one million-fold higher than the physiological levels. A number of studies have showed that melatonin possesses an antitumor effect on certain cancer types, including glioma and solid tumors with brain metastases [1,2,38,39]. In this study, melatonin also displayed anti-migratory and anti-invasive properties to U251 glioma cells under hypoxia. Importantly, our results showed that melatonin caused down-regulation of ROS production in U251 glioma cells under hypoxia and that hypoxia-induced migration and invasion were partially restrained via blocking elevated ROS by melatonin, which is similar to other ROS inhibitors. All of these results provide cues that the inhibitory effect of melatonin on glioma cell migration and invasion may partially result from its antioxidant effects.

To explore the underlying mechanisms of the above effect, ROS-related signaling was investigated. ROS, which are highly reactive O_2_ metabolites, serve as signaling molecules or directly oxidize important cellular proteins. Chronic and sustained generation of ROS can activate epithelial mesenchymal transition-related and metastasis-related genes including integrins [40-42]. Integrins play an important role in mediating cell-matrix and cell-cell interactions that have impacts on cell survival, proliferation, adhesion, migration and invasion. Expression of αvβ3 integrin correlates with the invasion and metastasis of several tumor types, including glioma, breast cancer, and melanoma [43,44]. Moreover, ROS accumulation could markedly up-regulate the expression of integrin αvβ3 heterodimers on the surface of colorectal cancer cells, which in turn promoted an aggressive phenotype in colorectal cancer cells [13,45]. The reduction of breast cancer cell adhesive affinity was also correlated with a down-regulation of ROS production and surface expression of activated integrin [46]. In the current study, we first found that hypoxia could significantly increase the ROS level and expression of αvβ3 integrin in U251 glioma cells compared with normoxia, while inhibition of ROS production by apocynin down-regulated the αvβ3 integrin expression. It was indicated that the expression of αvβ3 integrin can be up-regulated via ROS in U251 glioma cells under hypoxia. In addition, αvβ3 integrin knockdown also strongly inhibited the migration and invasion of U251 glioma cells under hypoxia. These results indicated that ROS-αvβ3 integrin signaling might be involved in hypoxia-induced migration and invasion of glioma cells. Because there was no report about the relationship between melatonin and αvβ3 integrin, these observations prompted us to explore the possible effect of melatonin on ROS-αvβ3 integrin signaling in U251 glioma cells under hypoxia. In response to melatonin treatment, ROS was remarkably reduced and αvβ3 integrin protein expression was also consequently decreased. It suggests that the inhibitory effect of melatonin on αvβ3 integrin might be mediated by ROS in U251 glioma cells.

It is well known that ligand binding to integrins leads to integrin clustering and association with proteins, which then result in focal adhesion clusters and the recruitment of actin filaments [47,48]. Focal adhesion kinases, including FAK and Pyk2, are widely recognized as important proteins in this process. Cell migration and invasion depend on the recruitment of FAK and/or Pyk2 to the newly formed focal adhesion sites [16]. The activation of FAK and Pyk2 is followed by the phosphorylation of a variety of downstream effectors, resulting in cell migration [49]. Many malignant human tumors exhibit increased FAK expression and tyrosine phosphorylation, which correlated with the acquisition of an invasive cellular phenotype and increased tumor metastasis [50]. In particular, Pyk2 is highly enriched in the CNS, and significant co-expression of FAK and Pyk2 in astrocytomas has also been demonstrated [51,52]. Recently, Lee et al. reported that melatonin induced the phosphorylation of FAK in umbilical cord blood-mesenchymal stem cells [23]. Therefore, we investigated whether the inhibitory effect of melatonin on U251 glioma cell migration and invasion is associated with the modulation of FAK and Pyk2 activation. Immunoblot results showed that hypoxia exposure resulted in a significant increase in phosphorylation of FAK at Tyr397 and Pyk2 at Tyr402 accompanied by comparable total protein levels. Remarkably, specific siRNA of FAK and Pyk2 significantly inhibited U251 glioma cell migration and invasion under hypoxia. It suggested that hypoxia-induced invasion of U251 glioma cells might be correlated with the level of activated Pyk2 and FAK, but not as a consequence of increased levels of the total amount of Pyk2 and FAK protein. In addition, αvβ3 integrin knockdown suppressed Pyk2 and FAK phosphorylation in U251 glioma cells under hypoxia. This is consistent with the report of Skuli, which also showed that hypoxia stimulated the αvβ3 integrin pathways through FAK in human glioblastoma cell lines and inhibiting the αvβ3 integrin by siRNA significantly reduced the amount of phosphorylated FAK in hypoxic glioblastoma cells [53]. Furthermore, we observed that ROS inhibitor decreased the amount of Pyk2 and FAK phosphorylated form in U251 glioma cells under hypoxia. Interestingly, we found that melatonin treatment could suppress Pyk2 and FAK phosphorylation without any change of total Pyk2 and FAK level, which is different from the report of Lee [23]. Our results showed that modulation of the phosphorylation of Pyk2 and FAK might be involved in the inhibition of glioma cell migration and invasion by melatonin under hypoxia. Collectively these data establish that ROS-αvβ3 integrin-FAK/Pyk2 pathway is associated with the migration and invasion of glioma cells under hypoxia, and that melatonin exerts anti-migratory and anti-invasive effects on glioma cells in response to hypoxia via this pathway.

In present study, however, the effect of melatonin on U251 glioma cells migration and invasion via ROS-αvβ3 integrin-FAK/Pyk2 pathway was also observed under normoxia. This gives a hint that the role of melatonin and ROS-αvβ3 integrin-FAK/Pyk2 pathway in hypoxia is not unique. A previous study also showed that melatonin could suppress migration and invasion via inhibition of oxidative stress pathway in glioma cells under normoxia [1]. These results suggest that ROS signaling pathway occurs not only in hypoxia but also in normoxia. Therefore, the different or specific hypoxia-related mechanism promoting tumor cell migration and invasion should be profoundly investigated.

## Conclusion

In summary, the pharmacologic concentration (1 mM) of melatonin was shown to display a significant inhibitory effect on the migration and invasion of glioma cells under hypoxia. Additionally, the anti-migration and invasion effects of melatonin were closely linked to the reduction of increased ROS levels in glioma cells. Importantly, the inhibition of ROS-αvβ3 integrin-FAK/Pyk2 pathway was demonstrated to be involved in the effect of melatonin on glioma cells. Taken together, melatonin exerts anti-migratory and anti-invasive effects on glioma cells in response to hypoxia and normoxia via ROS-αvβ3 integrin-FAK/Pyk2 signaling pathway. The current research displays a novel antitumor mechanism of melatonin and provides evidence that melatonin may be a potential therapeutic molecule, especially as a part of a drug cocktail targeting the hypoxic microenvironment of glioma.

## Additional files

Additional file 1:
**Effect of melatonin on the phosphorylation of FAK and Pyk2 in U251 glioma cells under normoxia.** The total and phosphorylation levels of FAK and Pyk2 in U251 glioma cells were determined by western blot analysis after melatonin treatment with different concentrations for 12 hours. Additional file 2:
**Effect of melatonin on the expression of αvβ3 integrin in U251 glioma cells under normoxia.** Specific fluorescence index (SFI) was used to evaluate the expression of αvβ3 integrin determined by immunofluorescence after treatment with melatonin. **p* <0.05 *vs* control. Additional file 3:
**Effect of melatonin on intracellular ROS of U251 glioma cells under normoxia.** Flow cytometry analysis was used to measure the level of intracellular ROS after treatment with melatonin. **p* <0.05 *vs* control.

## Abbreviations

FAK: Focal adhesion kinase
Pyk2: Proline-rich tyrosine kinase 2
ROS: Reactive oxygen species
ECM: Extracellular matrix
CNS: Central nervous system
FBS: fetAl bovine serum
siRNA: Small interfering RNA
