# Supplementary figures and images for: Involvement of ROS-alpha v beta 3 integrin-FAK/Pyk2 in the inhibitory effect of melatonin on U251 glioma cell migration and invasion under hypoxia
Source: J Transl Med. 2015 Mar 20;13:95. doi: 10.1186/s12967-015-0454-8 (PMC4371719; doi:10.1186/s12967-015-0454-8)

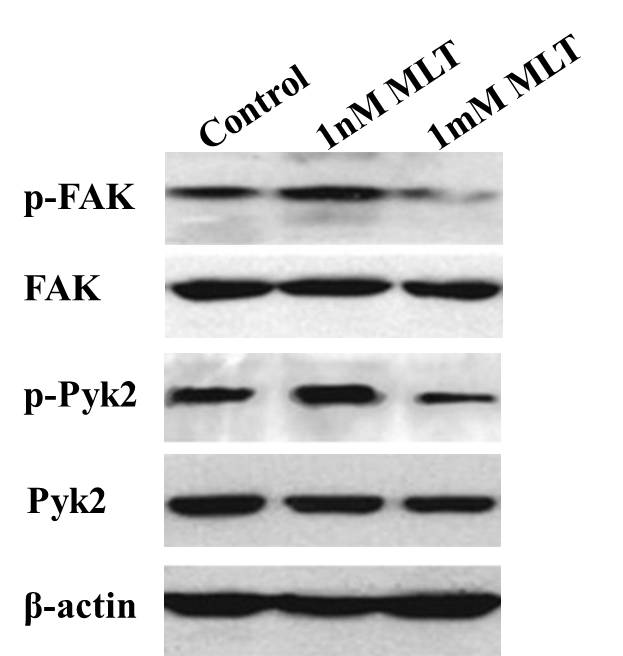

Supplement: Additional file 1: — Effect of melatonin on the phosphorylation of FAK and Pyk2 in U251 glioma cells under normoxia. The total and phosphorylation levels of FAK and Pyk2 in U251 glioma cells were determined by western blot analysis after melatonin treatment with different concentrations for 12 hours. [file 12967_2015_454_MOESM1_ESM.jpeg]

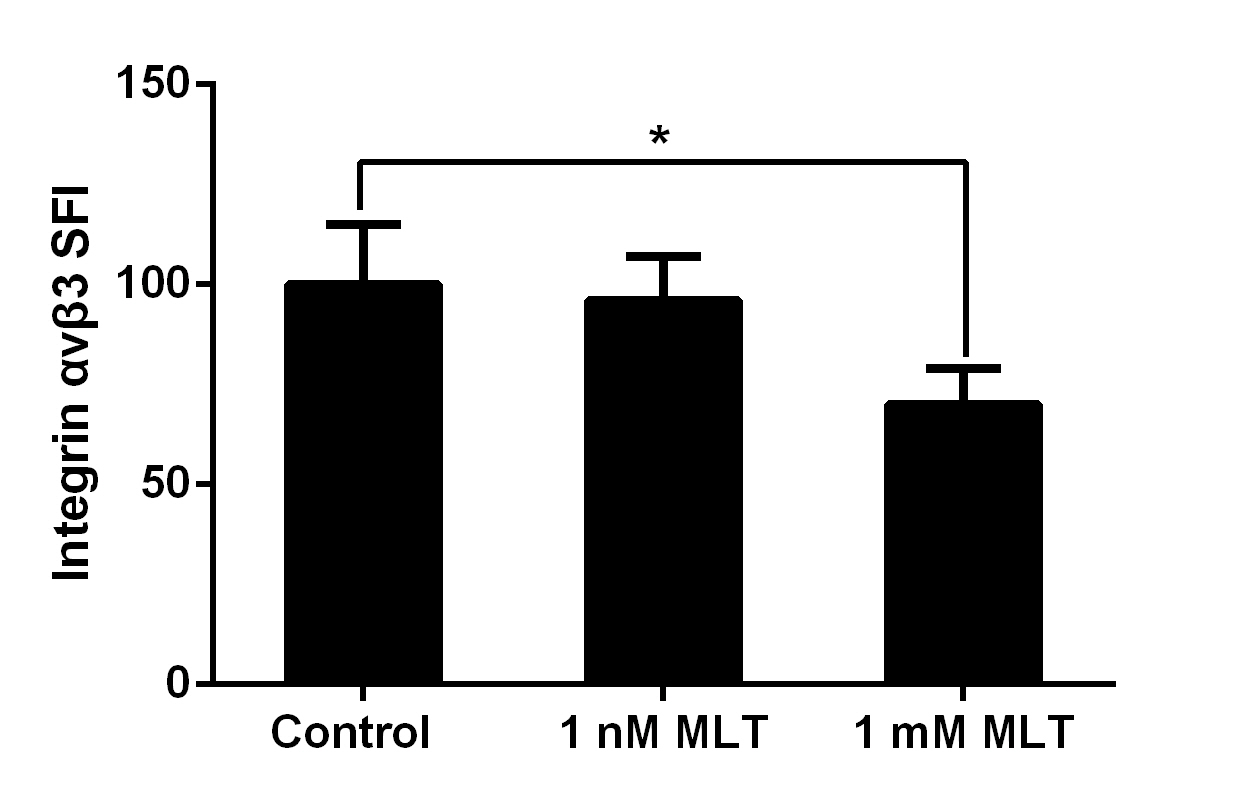

Supplement: Additional file 2: — Effect of melatonin on the expression of αvβ3 integrin in U251 glioma cells under normoxia. Specific fluorescence index (SFI) was used to evaluate the expression of αvβ3 integrin determined by immunofluorescence after treatment with melatonin. *p <0.05 vs control. [file 12967_2015_454_MOESM2_ESM.jpeg]

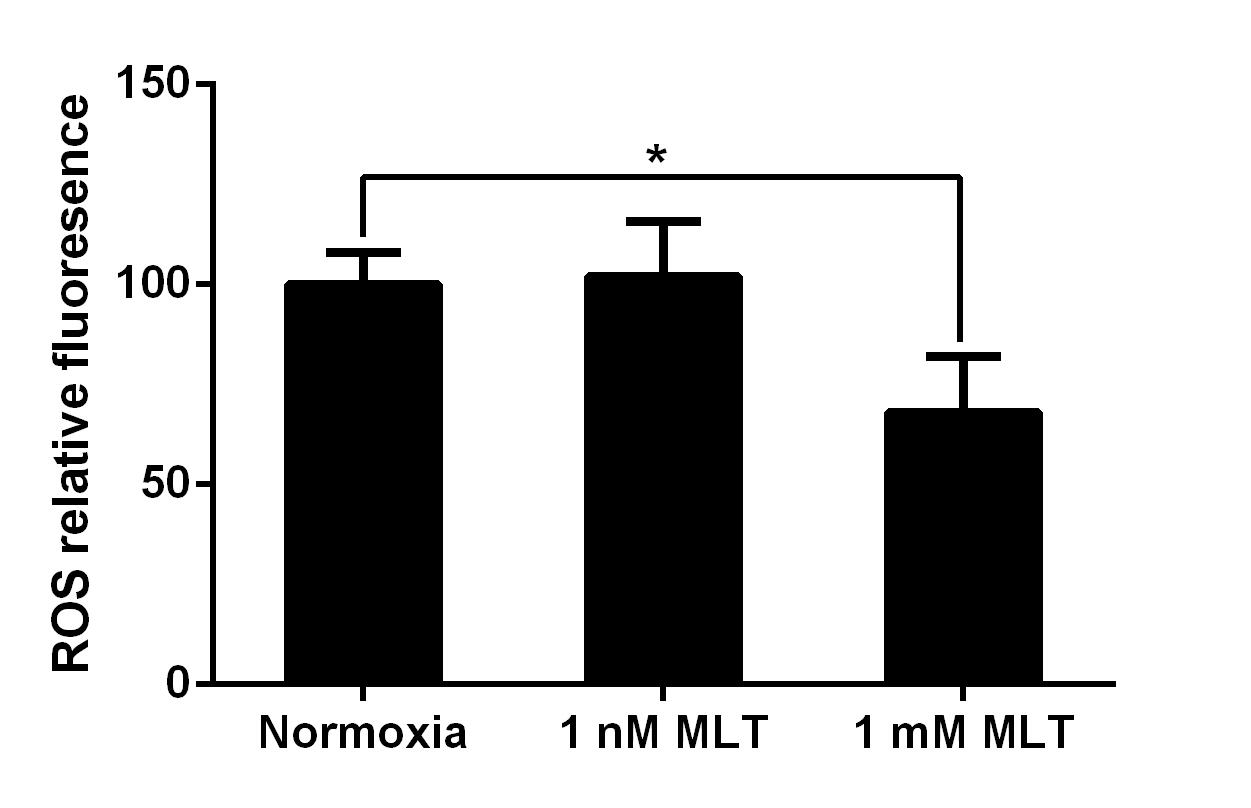

Supplement: Additional file 3: — Effect of melatonin on intracellular ROS of U251 glioma cells under normoxia. Flow cytometry analysis was used to measure the level of intracellular ROS after treatment with melatonin. *p <0.05 vs control. [file 12967_2015_454_MOESM3_ESM.jpeg]
